# Exchange Transfusion in the Treatment of Neonatal Septic Shock: A Ten-Year Experience in a Neonatal Intensive Care Unit

**DOI:** 10.3390/ijms17050695

**Published:** 2016-05-09

**Authors:** Lorenza Pugni, Andrea Ronchi, Bianca Bizzarri, Dario Consonni, Carlo Pietrasanta, Beatrice Ghirardi, Monica Fumagalli, Stefano Ghirardello, Fabio Mosca

**Affiliations:** 1 Neonatal Intensive Care Unit, Department of Clinical Sciences and Community Health, Fondazione IRCCS Ca’ Granda Ospedale Maggiore Policlinico, University of Milan, Via della Commenda 12, 20122 Milan, Italy; andrea.ronchi@mangiagalli.it (A.R.); carlo.pietrasanta@gmail.com (C.P.); beaghiro@hotmail.com (B.G.); monica.fumagalli@mangiagalli.it (M.F.); stefano.ghirardello@mangiagalli.it (S.G.); fabio.mosca@unimi.it (F.M.); 2 Neonatal Intensive Care Unit, Department of Pediatrics and Child Neuropsychiatry, “Sapienza” University of Rome, Viale del Policlinico 155, 00161 Rome, Italy; bizzarribianca@gmail.com; 3 Epidemiology Unit, Department of Preventive Medicine, Fondazione IRCCS Ca’ Granda Ospedale Maggiore Policlinico, University of Milan, Via della Commenda 12, 20122 Milan, Italy; dario.consonni@unimi.it

**Keywords:** neonate, preterm infant, neonatal infection, neonatal sepsis, neonatal septic shock, therapy of septic shock, exchange transfusion

## Abstract

Septic shock, occurring in about 1% of neonates hospitalized in neonatal intensive care unit (NICU), is a major cause of death in the neonatal period. In the 1980s and 90s, exchange transfusion (ET) was reported by some authors to be effective in the treatment of neonatal sepsis and septic shock. The main aim of this retrospective study was to compare the mortality rate of neonates with septic shock treated only with standard care therapy (ScT group) with the mortality rate of those treated with ScT and ET (ET group). All neonates with septic shock admitted to our NICU from 2005 to 2015 were included in the study. Overall, 101/9030 (1.1%) neonates had septic shock. Fifty neonates out of 101 (49.5%) received one or more ETs. The mortality rate was 36% in the ET group and 51% in the ScT group (*p* = 0.16). At multivariate logistic regression analysis, controlling for potentially confounding factors significantly associated with death (gestational age, serum lactate, inotropic drugs, oligoanuria), ET showed a marked protective effect (Odds Ratio 0.21, 95% Confidence Interval: 0.06–0.71; *p* = 0.01). The lack of observed adverse events should encourage the use of this procedure in the treatment of neonates with septic shock.

## 1. Introduction

Sepsis is still a major cause of morbidity and mortality in neonates, especially in preterm infants, causing approximately 36% of the estimated 4 million neonatal deaths annually [1,2]. Mortality rate can reach 60% in very low birth weight infants (VLBWI, birth weight < 1500 g) [2]. Early diagnosis, timely administration of appropriate antibiotics, and a proper supportive therapy are crucial to improve survival and to reduce long-term sequelae [3,4]. Unfortunately, neonatal sepsis can progress rapidly to septic shock, occurring in 1.3% of neonates hospitalized in neonatal intensive care unit (NICU), with an overall mortality of 40%, reaching 71% in neonates weighing less than 1000 g at the onset of sepsis [5].

Being aware of the persistently high mortality rate associated with sepsis and septic shock, despite an imposing and growing armamentarium of potent antibiotics, various adjunctive therapies like intravenous immunoglobulins and granulocyte-macrophage colony stimulating factors were evaluated in the treatment of sepsis and septic shock in the last decades. However, none of these strategies has been demonstrated to be able to reduce the mortality rate in sepsis and septic shock [6,7,8].

Case reports published in the medical literature in the 1970s [9,10] reporting the effective use of exchange transfusion (ET) in severe neonatal infection with sclerema prompted some authors to use this procedure as rescue therapy in neonates with severe sepsis in subsequent years [11,12,13]. The rationale for the use of ET using fresh, whole, adult blood is to remove bacteria, bacterial toxins, and circulating pro-inflammatory cytokines; to improve perfusion and tissue oxygenation; to correct the plasma coagulation system; and to enhance immunological defense mechanisms (increase in circulating levels of C3, immunoglobulins, improvement in the opsonic activity against the pathogen, enhancement of neutrophil function) [6,14,15,16,17,18,19,20].

Despite these potential benefits, very few studies were conducted in the last few decades to investigate the clinical efficacy of ET in neonatal sepsis and septic shock [16,20,21,22,23,24,25]. Although most studies showed some beneficial effects to the use of ET, clear evidence for its clinical efficacy is lacking. The discrepancy observed across studies can be attributed largely to the use of different inclusion and exclusion criteria, diagnostic criteria, and study designs.

Given the paucity of published data, we decided to perform a rigorous retrospective study on a large group of neonates in order to compare the mortality rate of neonates with septic shock treated only with standard care therapy (*i.e.*, antibiotics, respiratory and cardiocirculatory support therapy) (ScT group) with those treated with ScT and ET (ET group).

## 2. Results

Of the 9030 neonates admitted to the NICU during the study period, 101 (1.1%) met the inclusion and exclusion criteria of the study. Eighty-nine of the 101 neonates (88%) were born prematurely, and 51 (50%) were extremely preterm infants (<28 weeks’ gestation).

Out of 101 neonates, 50 (49.5%) received one or more ETs for a total of 79 ETs: 28/50 neonates (56%) received one ET, 17/50 neonates (34%) received two ETs, 4/50 neonates (8%) received three ETs, and 1/50 neonates (2%) received five ETs. ETs were performed through an umbilical venous or arterial catheter in most neonates (44/50), and through a central venous catheter placed in the internal jugular vein or in the femoral vein in the remaining six neonates.

Table 1 shows the characteristics of the study population. The median GA of neonates in the ET group was significantly higher than in the ScT group (28 weeks, IQR 26–31, *vs.* 26 weeks, IQR 24–29; *p* = 0.05). The median birth weight was also significantly higher among neonates who underwent ET (1060 g, IQR 770–2000, *vs.* 750 g, IQR 580–1100; *p* = 0.003). The proportion of extremely low birth weight infants (birth weight < 1000 g) was significantly lower in the ET group (46% *vs.* 72.5%; *p* = 0.008).

As shown in Table 1, EOS occurred in 27/50 neonates (54%) in ET group and in 14/51 neonates (27.4%) in ScT group, while LOS occurred in 23/50 neonates (46%) in ET group and in 37/51 (72.5%) in ScT group (*p* = 0.008). Median age at sepsis evaluation was significantly lower in ET group than in ScT group (2 days, IQR 0–11, *vs.* 9 days, IQR 3–22; *p* = 0.003). On the contrary, the median time between the onset of sepsis and the appearance of septic shock was significantly shorter in ET group than in ScT group (4.5 h, IQR 3–10, *vs.* 13 h, IQR 5–29; *p* = 0.001).

Out of 101 neonates, 95 underwent a blood culture before antibiotic administration (47 in ET group and 48 in ScT group), 14 underwent a lumbar puncture, and five had a urine culture. Blood culture was positive in approximately half the cases in both groups. In Table 2, the pathogens cultured from sterile sites in the population studied are reported.

Table 3 compares clinical signs and symptoms at the appearance of septic shock between the two groups studied. Level of sickness assessed with the SNAP-II score was comparable between the groups (median score 32, IQR 19–50, in ET group *vs.* 41, IQR 25–50, in ScT group; *p* = 0.32). Respiratory signs (apnea, tachypnea) were present in more than 90% of neonates and hypotension in more than 80% of neonates in both groups, whereas tachycardia was more frequent in the ET group than in the ScT group (90% *vs.* 68.6%; *p* = 0.01).

All neonates underwent a diagnostic laboratory evaluation. In Table 4, laboratory results are shown. Neonates in ET group had a significantly lower number of white blood cells than neonates in ScT group (median 4750/mm^3^, IQR 2890–11,530, *vs.* 9540/mm^3^, IQR 5620–20,100; *p* = 0.0006), as well as a significantly lower number of neutrophils (median 1690/mm^3^, IQR 980–5400, *vs.* 4150/mm^3^, IQR 2050–8320; *p* = 0.01). Neonates in ET group had also a PT ratio significantly longer (1.88, IQR 1.62–2.38, *vs.* 1.64, IQR 1.25–1.91; *p* = 0.05).

All infants included in the study received respiratory and circulatory support at the onset of septic shock, as shown in Table 5. The proportion of neonates treated with dobutamine, hydrocortisone, and epinephrine was higher in ET group than in ScT group. Neonates in ET group received higher doses of dopamine (median dose 10 mcg/kg/min, IQR 10–15, *vs.* 7.5, IQR 5–15; *p* = 0.05), dobutamine (median dose 10 mcg/kg/min, IQR 7.5–15, *vs.* 5, IQR 5–10; *p* = 0.01), hydrocortisone (0.3 mg/kg/hour, IQR 0.2–0.3, *vs.* 0.1, IQR 0.1–0.2; *p* = 0.05).

In the ET group, laboratory values were monitored before and after the procedure. As shown in Table 6, the median value of white blood cells significantly increased after the procedure (10,630/mm^3^ *vs.* 4750/mm^3^; *p* = 0.02), while the median value of platelets significantly decreased (37,500/mm^3^ *vs.* 87,500/mm^3^; *p* < 0.001), and 29 neonates (58%) required a platelet transfusion after ET. After ET, base excess significantly decreased from −8.8 to −5.2 mmol/L (*p* < 0.001) and urine output significantly increased. All the neonates undergoing ET received glucose during the procedure to maintain glycemia in a normal range; none of them had hypoglycemia during the procedure. None of the neonates undergoing ET had hypocalcemia during or after the procedure.

No serious adverse events were attributable to the ET procedure. Two out of 50 patients (2%) died during ET. However, both patients were moribund before starting the procedure, with elevated serum lactate (>20 mmol/L) and a need for a high dose of inotropic drugs. The first infant had an EOS with negative blood culture; the other one had a LOS caused by *K. pneumoniae*. Two out of 41 (4.8%) patients with normal cerebral ultrasound before ET showed a 1st–2nd grade intraventricular hemorrhage after the procedure.

Overall, 44 out of 101 neonates (43.5%) died because of septic shock. The rate of sepsis-related death was lower in the ET group than in the ScT group (36% *vs.* 51%; *p* = 0.16), even if the difference did not reach a statistical significance. The median age at time of death was higher in the ScT group than in the ET group (13 days, IQR 7.5–44, *vs.* 5 days, IQR 1–18; *p* = 0.01).

**Figure 1:**
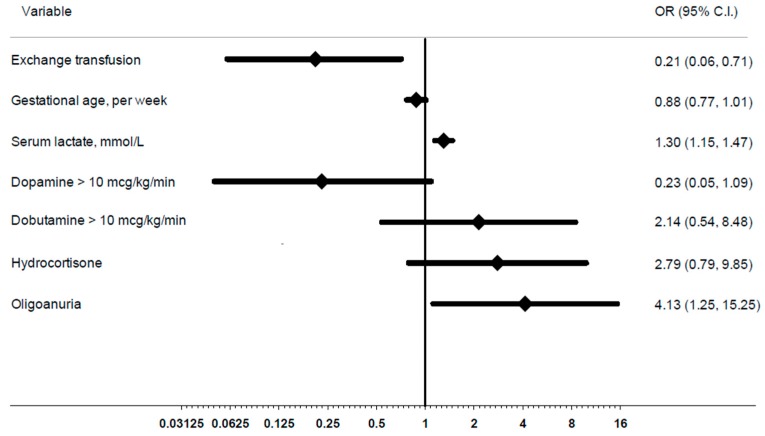
Adjusted odds ratios (ORs) (diamond) of death and 95% confidence intervals (CI) (bold line) in neonates who underwent exchange transfusion in comparison with the standard care therapy and according to selected clinical variables. Each variable is adjusted for the others.

The crude OR (95% CI) of death in the ET group was 0.54 (0.24–1.91). However, at multivariate logistic regression analysis, controlling for potentially confounding factors (GA, serum lactate, inotropic drugs, oligoanuria), ET showed a marked protective effect (OR 0.21, 95% CI: 0.06–0.71; *p* = 0.01) (Figure 1). Even adjusting for birth weight, weight, and age at sepsis evaluation, ORs were almost identical.

## 3. Discussion

Sepsis is a life-threatening condition and continues to be a major challenge for physicians, especially in intensive care units. In the neonatal period, the disease runs a rapid course to septic shock, occurring in 1.3% of neonates hospitalized in the NICU with an overall mortality of 40%, reaching 71% in neonates weighing less than 1000 g at the onset of the disease [5].

In the last decades, particularly in the 80s and 90s, ET has been proposed as an adjunctive therapy for severely septic neonates [9,10,11,12,13,16,20,22,23,24,25]. However, despite its potential benefits, clear evidence for its clinical efficacy is lacking, as most reports were anecdotal or conducted on small groups of neonates without comparative controls.

Given the paucity of published data, the main aim of this retrospective study conducted on a large group of neonates was to compare the mortality rate of neonates with septic shock treated only with ScT (ScT group) with those treated with ScT and ET (ET group).

During the 10-year study period, 1.1% of neonates admitted to our NICU had septic shock, defined rigorously in accordance with Goldstein’s and Wynn’s criteria. The incidence of septic shock we found was similar to that reported by Kermorvant-Duchemin *et al.* in 2008 [5].

Overall, the mortality rate associated with septic shock we found was 43.5%, similar to that reported by Kermorvant-Duchemin in the aforementioned study [5]. The mortality rate was lower in the ET group than in the ScT group (36% *vs.* 51%), even if the difference did not reach statistical significance through univariate analysis. Nevertheless, the subsequent multivariate logistic regression analysis showed that the variables that increased the risk of death were the high level of serum lactate and the presence of oliguria and anuria, while receiving ET reduced the risk of mortality by 80%.

The reduction of mortality rate among infants with severe sepsis and septic shock undergoing ET was already reported by other authors. To date, only three randomized controlled trials have been published. In 1993, Mathur *et al.* [20] conducted a randomized controlled trial in neutropenic septic neonates, having as their primary objective the evaluation of neutrophilic function after ET; they reported a mortality rate of 35% (7 out of 20) in the ET group and 70% (7 out of 10) in the control group (*p* = 0.07). In 1997, Sadana *et al.* [16] randomized culture-positive septic neonates with sclerema to undergo ET or not. The authors reported a mortality rate of 50% (10 out of 20) in the study group and 95% (19 out of 20) in the controls (*p* = 0.003). IgG, IgA, and IgM rose significantly after ET, but complement (C3) levels did not change. Very recently, Aradhya *et al.* [25] performed a randomized controlled trial to demonstrate the efficacy of ET in improving the outcome of neonates weighing more than 1000 g at birth with severe sepsis. The neonates were randomized to receive standard therapy alone or standard therapy with ET. Authors concluded that ET showed a trend towards reduction in mortality of 21% in comparison to standard therapy. However, the study was terminated without reaching the calculated sample size. Beyond these few randomized controlled trials, the majority of published studies on this topic are scarce, dated, conducted on few cases, using different inclusion and exclusion criteria, different and non-rigorous definitions of septic shock, and not having as their primary objective the mortality rate. Although most studies showed some beneficial effect with the use of ET, some authors reported different results. In 1981, Bossi *et al.* [21] described their experience that included 35 neonates with severe sepsis, 22 of them treated with ET, and 13 with standard therapy alone. The survival rate was similar between the two groups (ET, 54.5% *vs.* ScT, 53.8%; *p* = No Significant). A controlled trial [26], which had as its primary outcome an evaluation of whether ET would improve the disseminated intravascular coagulation in neonates with septic shock, concluded that the mortality rate was the same among those who received ET and those who did not receive it.

The rationale for the use of ET using fresh, whole, adult blood is to remove bacteria, bacterial toxins, and circulating pro-inflammatory cytokines; to improve perfusion and tissue oxygenation; to correct the plasma coagulation system; and to enhance immunological defense mechanisms (increase in circulating levels of C3, immunoglobulins, improvement in the opsonic activity against the pathogen, enhancement of neutrophil function) [6,14,15,16,17,18,19,20].

In our study, we documented a correction of leukopenia and neutropenia, and an improvement of renal perfusion and in the acid base status after ET, which could in part explain, according to the medical literature, the protective effect of ET. In fact, in our patients treated with ET, the comparison between laboratory parameters before and after ET showed an increased number of white blood cells and neutrophils, and an improvement of urine output. These findings have already been described by other authors. Xanthou *et al.* [17] described 20 infants who received 27 ET for hyperbilirubinemia and found a significant increase of polymorphonuclear cells immediately after the procedure and 6 h later. Even Phibbs *et al.* [27] showed that after ET the values of polymorphonuclear cells reached pre-procedure values and even higher. In a randomized controlled trial that included 30 infants with proven sepsis and neutropenia, the absolute neutrophil count significantly increased 6 h after ET. The mechanism most likely to be able to increase significantly the number of neutrophils is movement of those attached to blood vessels and from the extravascular pool (believed to contain 20 times more than the circulating amount), stimulated by factors contained in the blood of the donor able to mobilize leukocytes [17]. An improvement of urine output after ET was already reported by Levinson *et al.* [28] in their retrospective study, in which they described four adult patients with septic shock who had an improvement in urine output after ET. A significant improvement of metabolic acidosis was reported by several authors [11,12,25] and was attributed to a better perfusion and tissue oxygenation.

In this study, the neonates treated with ET had an earlier onset of sepsis and a more rapid progression to septic shock. This fact can be attributed to a higher percentage of neonates, both term and preterm, with early-onset sepsis caused by Gram-negative pathogens in the ET group. Gram-negative organisms are very aggressive and able to cause sepsis rapidly evolving into septic shock, leading the neonatologist to choose ET. Overall, Gram-negative pathogens were the most common pathogens cultured from the sterile sites in the ET group. Considering the higher mortality rate for sepsis caused by Gram-negative pathogens reported in the literature [29], the most dramatic evolution of sepsis could be expected in the ET group. Leukopenia and neutropenia, more frequently observed in the ET group, could be attributed to the high incidence in this group of sepsis caused by Gram-negative pathogens, the most frequent cause of bone marrow depression strictly related to their virulence [30]. Furthermore, the neonates in the ET group had greater cardiovascular dysfunction. In fact, they had more frequent tachycardia at the onset of the disease and required higher doses of inotropic drugs and hydrocortisone.

No serious adverse events were attributable to the ET procedure in our study, according to the literature [31]. ET was introduced in the late 1940s in the treatment of hemolytic disease of the newborn. Subsequently, many other conditions, such as disseminated intravascular coagulation and neonatal sepsis, have been treated with this procedure in the NICUs [31]. The most commonly reported ET-related adverse effects are thrombocytopenia, hypocalcemia, hyperkalemia, apnea, bradycardia, hypotension, and catheter-related complications. In 2007, Steiner *et al.* [31] described ET-related morbidity and mortality over a 20-year period (1986–2006) at Yale New Haven Hospital. The authors reported that the majority of ET complications were transient, and serious adverse events occurred only in premature or very sick neonates. No deaths were related to ET during the study period.

In our study, about half of the neonates required a platelet transfusion after ET. Thrombocytopenia is one of the most common ET-related adverse effects reported in the literature. However, none of the neonates had bleeding related to the fall of platelets. None of the neonates had hypocalcemia during or after the procedure. Two out of 50 patients (2%) died during ET. However, both patients were moribund before starting the procedure.

The limitation of this study is its retrospective nature. Despite this limitation, it has to be stressed that the study was conducted over 10 years in the same NICU and the decision to perform the procedure has been made by the same medical team. In our opinion, the strengths of this study are represented by the fact that it was conducted on a large group of neonates, the inclusion criteria were strict, and the use of electronic charts allowed for precise data collection.

## 4. Material and Methods

### 4.1. Study Population

This was a retrospective single-center study conducted at the NICU of Fondazione IRCCS Ca’ Granda Ospedale Maggiore Policlinico of Milan, Italy, from March 2005 to March 2015.

The NICU of Fondazione IRCCS Ca’ Granda Ospedale Maggiore Policlinico of Milan is a 23-bed, level 3B NICU with approximately 800–900 admissions per year, 120–140 of whom are VLBWI.

All septic neonates hospitalized in the NICU who met Goldstein’s or Wynn’s criteria [32,33] for the diagnosis of septic shock in term and preterm neonates, respectively, were included in the study. Exclusion criteria were not meeting Goldstein’s or Wynn’s criteria, the presence of major congenital malformations, and lack of written informed parental consent to the ET procedure.

Eligible study subjects were identified by reviewing the NICU database and the International Classification of Diseases, 9th revision (ICD9) codes for sepsis and septic shock. Demographic, clinical, and laboratory data were recorded by means of neonates’ electronic charts.

The neonates included in the study were divided into two groups, depending on whether they received only standard care therapy (ScT group) or ET in addition to standard care therapy (ET group). The decision on the implementation of the procedure was always taken by the same medical team over the 10 years analyzed.

### 4.2. Definitions

Sepsis and septic shock were defined according to Goldstein’s or Wynn’s criteria [32,33] for term and preterm neonates, respectively. Term neonates were defined as those born at a gestational age (GA) of 37–42 weeks; preterm neonates were defined as those born at a GA less than 37 weeks. GA was established on the basis of best obstetric estimates, including last menstrual period and first or second trimester ultrasonography. Small for gestational age (SGA) neonates were defined as those with a birth weight less than the 10th centile for age, according to Bertino’s anthropometric charts [34]. Early-onset sepsis (EOS) and late-onset sepsis (LOS) were defined as the onset of clinical signs or symptoms consistent with sepsis at ≤72 or >72 h of life, respectively [1]. Hypothermia, hyperthermia, tachypnea, tachycardia, bradycardia, hypotension, poor refill time, and oliguria were defined according to Goldstein and Wynn [32,33]. Hypothermia was defined as core temperature <36 °C, and hyperthermia as core temperature >38 °C in term neonates and >38.5 °C in preterm neonates. Tachypnea was defined as mean respiratory rate >2 Standard Deviation (SD) above normal for age. Tachycardia was defined as mean heart rate >2 SD above normal for age, bradycardia as mean heart rate <10th percentile for age. Hypotension was defined as a decrease in blood pressure <5th percentile for age or systolic blood pressure >2 SD below normal for age. Capillary refill time was considered poor if >5 s in term neonates and >4 s in preterm neonates. Oliguria was defined by a urine output <0.5 mL/kg/h. Feeding intolerance was defined as gastric residual >50% of the volume of milk administered or vomiting and/or abdominal distension or reduction or discontinuation of feeding milk [35]. Intraventricular hemorrhage (IVH) was defined using Papile’s classification and included all grades detected by cranial ultrasound [36]. The severity of illness at the onset of septic shock was assessed using the revised version of the Score for Neonatal Acute Physiology (SNAP-II) [37]. Death was considered to be related to sepsis if it occurred within seven days of the onset of the disease or if clinical signs and symptoms of sepsis were believed to be the direct cause of death [38]. Death was considered related to ET if it occurred during the procedure or within 6 h. Side effects were attributed to the procedure if it happened during the procedure or within 6 h.

### 4.3. Data Collection

The following variables were considered in this study: GA, birth weight, weight for GA, birth place, number of fetuses, gender, Apgar score at 1 and 5 min, need for resuscitation at birth, age and weight at sepsis evaluation, time between sepsis and septic shock, time between septic shock and ET procedure, clinical signs and symptoms at the onset of septic shock (hyperthermia, hypothermia, apnea, tachypnea, tachycardia, bradycardia, hypotension, capillary refill, neurologic signs, urinary output, feeding intolerance, SNAP-II score), laboratory tests at the onset of septic shock (complete blood cell count with differential, C-reactive protein, coagulation tests, blood gas analysis, hepatic and kidney assessment), supportive therapy for septic shock (respiratory support, oxygen requirement, inhaled nitric oxide, intravenous fluid, inotropic drugs, hydrocortisone), pathogens cultured from sterile sites (blood, cerebrospinal fluid, urine), and age at time of death.

### 4.4. Exchange Transfusion (ET) Procedure

ET is a procedure by which an infant’s blood is replaced with donor blood by repeatedly removing and replacing small aliquots of blood over a short time period. For all neonates, we used fresh (collected <5 days before the procedure), filtered, 0 Rh-negative packed red blood cells suspended in AB plasma in a ratio to ensure a hematocrit of 4%–60%. Aliquots of 5–10 mL of donor blood were exchanged with infant blood until a cumulative volume of exchange of 160 mL/kg (double the estimated infant blood volume) was reached. In order to prevent hypocalcemia, we administered 1 mL/kg of 10% calcium gluconate after 100 mL of blood exchange. ET was always performed using an umbilical venous or arterial catheter or a central venous catheter (double-lumen) placed in the internal jugular vein or in the femoral vein by the surgical cutdown or Seldinger technique. The “pull–push technique” through a special four-way stopcock was used. The procedure was completed within 90–150 min [39,40].

### 4.5. Statistical Analyses

Median and interquartile range (IQR) were used to describe quantitative variables. Proportions were calculated for categorical variables. We calculated chi-squared and Fisher’s exact test (for categorical variables) or Mann–Whitney *U* test (for continuous variables) for comparisons between ET and ScT groups. For before and after comparisons, we used the Stuart–Maxwell test (similar to the McNemar test but suitable for variables with more than two categories) for categorical variables and the Wilcoxon signed-ranks test for quantitative variables. To assess the risk of death we used univariate and multiple logistic regression models to calculate odds ratios (ORs) and 95% confidence interval (95% CI). In multiple logistic models we included as potential confounders a set of selected variables considered *a priori* clinically relevant. Statistical analysis was performed using Stata 13 (StataCorp. 2013. Stata: Release 13 Statistical Software. Stata Corp LP: College Station, TX, USA).

## 5. Conclusions

In conclusion, a significant reduction of mortality in patients who underwent ET, together with the lack of adverse effects observed, suggest that this procedure should be considered for the treatment of neonates with septic shock.

## Figures and Tables

**Table 1:** Characteristics of the study population.

Clinical Variables	ET (*n* = 50)	ScT (*n* = 51)	*p* Value *
Gestational age, wks, median (IQR)	28 (26–31)	26 (24–29)	**0.05**
Birth weight, g, median (IQR)	1060 (770–2000)	750 (580–1100)	**0.003**
≤1000 g, *n* (%)	23 (46)	37 (72.5)	**0.008**
1001–1500 g, *n* (%)	11 (22)	5 (5.8)	0.11
>1500 g, *n* (%)	16 (32)	9 (17.6)	0.11
SGA, *n* (%)	5 (10)	13 (25.5)	0.07
Inborn, *n* (%)	44 (88)	44 (86.3)	1
Twin, *n* (%)	14 (28)	11 (21.6)	0.49
Male, *n* (%)	31 (62)	33 (64.7)	0.83
Apgar 1 min, median (IQR)	5 (2–7)	5 (2–6)	0.43
Apgar 5 min, median (IQR)	8 (7–8)	7 (7–8)	0.66
Resuscitation in delivery room, *n* (%) ^1^	42 (84)	45 (88.2)	0.57
Early-onset sepsis, *n* (%)	27	14	**0.008**
Late-onset sepsis, *n* (%)	23	37	**0.008**
Age at sepsis evaluation, d, median (IQR)	2 (0–11)	9 (3–22)	**0.003**
Weight at sepsis evaluation, g, median (IQR)	1212.5 (820–1960)	880 (600–1610)	**0.05**
Time between sepsis and septic shock, h, median (IQR)	4.5 (3–10)	13 (5–29)	**0.001**
Time between septic shock and ET, h, median (IQR)	7 (4–18)	NA	NA

* *Chi*-squared test for categorical variables, Mann–Whitney *U* test for quantitative variables; ET: Exchange transfusion; ScT: Standard care therapy; IQR: Interquartile range; SGA: Small for gestational age; (^1^) at least ventilation with mask. Bold number: *p* Value ≤ 0.05; NA: not applicable.

**Table 2:** Distribution of pathogens cultured from sterile sites.

Cultures	ET	ScT	*p* Value *
**Blood culture, *n***	47	48	
**Any bacteria, *n* (%)**	26 (55.3)	27 (56.2)	1
**Gram-positive organisms, *n* (%)**	5 (10.6)	15 (31.2)	**0.02**
*S. agalactiae*, *n* (%)	1 (2)	2 (3.9)	
*S. aureus*, *n* (%)	0	1 (1.9)	
*S. aureus* MRSA, *n* (%)	0	2 (3.9)	
*S. aureus* MSSA, *n* (%)	1 (2)	2 (3.9)	
*S. epidermidis*, *n* (%)	3 (6)	5 (9.8)	
*E. faecalis*, *n* (%)	0	1 (1.9)	
*E. faecium*, *n* (%)	0	1 (1.9)	
Others, *n* (%)	0	1 (1.9)	
**Gram-negative organisms, *n* (%)**	21 (44.6)	12 (25)	**0.05**
*E. coli*, *n* (%)	3 (6)	0	
*E. coli* ESBL neg, *n* (%)	2 (4)	3 (5.8)	
*E. coli* ESBL pos, *n* (%)	1 (2)	0	
*E. cloacae*, *n* (%)	1 (2)	1 (1.9)	
*K. pneumoniae*, *n* (%)	3 (6)	0	
*K. pneumoniae* ESBL pos, *n* (%)	3 (6)	2 (3.9)	
*K. pneumoniae* ESBL neg, *n* (%)	0	1 (1.9)	
*P. aeruginosa*, *n* (%)	7 (14)	3 (5.8)	
*S. marcescens*, *n* (%)	1 (2)	0	
*S. maltophilia*, *n* (%)	0	1 (1.9)	
*M. morganii*, *n* (%)	0	1 (1.9)	
**Fungi, *n* (%)**	2 (4)	0	0.24
*C. albicans*, *n* (%)	2 (4)	0	
**Spinal fluid culture, *n***	4	10	–
**Any bacteria, *n* (%)**	2 (50)	2 (20)	0.8
*S. marcescens*, *n* (%)	1 (25)	0	
*S. agalactiae*, *n* (%)	1 (25)	1 (10)	
*E. coli*, *n* (%)	0	1 (10)	
**Urine culture, *n***	4	1	0.24
*P. aeruginosa*, *n* (%)	1 (25)	0	

* *Chi*-squared test for categorical variables; ET: Exchange transfusion; ScT: Standard care therapy; MRSA: Methicillin-resistant *Staphylococcus aureus*; MSSA: Methicillin-sensitive *Staphylococcus aureus*; ESBL: Extended-spectrum beta-lactamase. Bold number: *p* Value ≤ 0.05.

**Table 3:** Clinical characteristics of the study population at the onset of septic shock.

Clinical Signs/Symptoms	ET (*n* = 50)	ScT (*n* = 51)	*p* Value *
**Hyperthermia, *n* (%)**	6 (12)	5 (9.8)	0.76
**Hypothermia, *n* (%)**	6 (12)	11 (21.6)	0.28
**Respiratory signs, *n* (%)**	–	–	–
Any, *n* (%)	48 (96)	50 (98)	0.6
Apnea, *n* (%)	32 (62.7)	32 (64)	1
Tachypnea, *n* (%)	48 (96)	49 (96.1)	1
**Feeding intolerance, *n* (%)**	16 (32)	29 (56.9)	**0.01**
**Tachycardia, *n* (%)**	45 (90)	35 (68.6)	**0.01**
**Hypotension, *n* (%)**	42 (84)	45 (88.2)	0.57
**Poor capillary refill, *n* (%)**	45 (90)	48 (94.1)	0.48
**Neurologic signs, *n* (%)**	–	–	–
Any, *n* (%)	36 (72)	40 (78.4)	0.49
Hypotonia, *n* (%)	28 (56)	36 (70.6)	0.15
Lethargy, *n* (%)	28 (56)	40 (78.4)	**0.02**
Seizures, *n* (%)	2 (3.9)	2 (4)	1
**Diuresis**	–	–	–
Oliguria/anuria, *n* (%)	24 (48)	15 (29)	0.14
Oliguria, *n* (%)	7 (14)	4 (8)	0.35
Anuria, *n* (%)	17 (34)	11 (21)	0.19
**SNAP-II, score, median (IQR)**	32 (19–50)	41 (25–50)	0.32
<20	13	8	–
20–40	15	15	–
>40	22	28	–

* *Chi*-squared test for categorical variables, Mann–Whitney *U* test for quantitative variables; ET: Exchange transfusion; ScT: Standard care therapy; SNAP-II: Score for Neonatal Acute Physiology-Version II; IQR: Interquartile range. Bold number: *p* Value ≤ 0.05.

**Table 4:** Laboratory evaluation in the study population at the onset of septic shock.

Laboratory Data	ET (*n* = 50)	ScT (*n* = 51)	*p* Value *
**Complete Blood Cell Count**			
White cell blood count, cells/mm^3^, median (IQR)	4750 (2890–11,530)	9540 (5620–20,100)	**0.0006**
Neutrophils, cells/mm^3^, median (IQR)	1690 (980–5400)	4150 (2050–8320)	**0.01**
Platelets, *n*/mm^3^, median (IQR)	87,500 (37,500–160,000)	130,000 (45,000–250,000)	0.18
**C-reactive protein, mg/dL, median (IQR)**	4.71 (0.90–10.13)	2.65 (1–6.5)	0.56
**Coagulation Tests**			
PT ratio, median (IQR)	1.88 (1.62–2.38)	1.64 (1.25–1.91)	**0.05**
APTT ratio, median (IQR)	1.88 (1.64–2.73)	1.95 (1.4–5)	0.86
Fibrinogen, mg/dL, median (IQR)	202.5 (161–278)	233 (199–334)	0.34
**Blood Gas Analysis**			
Base excess, mmol/L, median (IQR)	−8.8 (−13.1, −5.75)	-8.1 (−13.1, −3.1)	0.68
Serum lactate, mmol/L, median (IQR)	6.95 (3–12.6)	5.3 (2.5–8.9)	0.2
**Hepatic Assessment**			
Serum alanine aminotransferase, U/L, median (IQR)	11 (6–20)	8 (5–15)	0.4
Serum aspartate aminotransferase, U/L, median (IQR)	46 (32–80)	42.5 (24–72)	0.36
**Kidney Assessment**			
Serum creatinine, U/L, median (IQR)	0.7 (0.6–1)	0.9 (0.7–1.12)	0.09
Serum urea, U/L, median (IQR)	49 (35–65)	56 (35–83)	0.19

* *Chi*-squared test for categorical variables, Mann–Whitney *U* test for quantitative variables; ET: Exchange transfusion; ScT: Standard care therapy; IQR: Interquartile range. Bold number: *p* Value ≤ 0.05.

**Table 5:** Supportive therapy for the study population at the onset of septic shock.

Supportive Therapy	ET (*n* = 50)	ScT (*n* = 51)	*p* Value *
**Respiratory Support**			
Any ventilation, *n* (%)	50 (100)	51 (100)	1
Non-invasive ventilation, *n* (%)	3 (6)	2 (4)	0.67
Mechanical ventilation, *n* (%)	21 (42)	31 (61)	0.07
High frequency oscillatory ventilation, *n* (%)	26 (52)	18 (35)	0.11
**Oxygen requirement, *n* (%)**	46 (92)	49 (96)	0.4
**Inhaled nitric oxide, *n* (%)**	26 (52)	21 (37)	0.3
**Intravenous Fluid, *n* (%)**			
Any fluid, *n* (%)	49 (98)	49 (96)	–
Saline bolus, *n* (%)	45 (90)	44 (86)	0.75
Plasma bolus, *n* (%)	27 (54)	31 (61)	0.55
**Inotropic Agents**			
Any inotropic agents, *n* (%)	42 (84)	45 (88)	0.6
Dopamine, *n* (%)	40 (80)	41 (80)	1
Dobutamine, *n* (%)	35 (70)	29 (57)	0.2
Epinephrine, *n* (%)	9 (18)	2 (4)	**0.03**
**Hydrocortisone, *n* (%)**	25 (50)	16 (31)	0.07

* *Chi*-squared test for categorical variables, Mann–Whitney *U* test for quantitative variables; ET: Exchange transfusion; ScT: Standard care therapy. Bold number: *p* Value ≤ 0.05.

**Table 6:** Laboratory evaluation before and after exchange transfusion (ET).

Laboratory Data	Pre-ET (*n* = 50)	Post-ET (*n* = 51)	*p* Value *
**Complete Blood Cell Count**			
White cell blood count, cells/mm^3^, median (IQR)	4750 (2890–11,530)	10,630 (6152–16,290)	**0.02**
Neutrophils, cells/mm^3^, median (IQR)	1690 (980–5095)	5130 (3245–8665)	0.08
Platelets, *n*/mm^3^, median (IQR)	87,500 (38,700–155,500)	37,500 (14,500–61,200)	**<0.001**
**C-reactive Protein, mg/dL, Median (IQR)**	4.71 (0.9–10.13)	5.52 (1.04–12.03)	0.18
**Blood Gas Analysis**			
Base excess, mmol/L, median (IQR)	−8.8 (−13.1, −5.7)	−5.2 (−8.5, −3.2)	**<0.001**
Serum lactate, mmol/L, median (IQR)	6.95 (3–12.4)	5.6 (2.6–12.6)	0.88
**Kidney Assessment**			
Serum creatinine, U/L, median (IQR)	0.75 (0.6–1.02)	0.95 (0.6–1.22)	0.22
Serum urea, U/L, median (IQR)	49 (35.5–64.5)	78 (43–101)	**0.0002**

* Wilcoxon signed-ranks test for quantitative variables, Stuart–Maxwell test for categorical variables; ET: Exchange transfusion; ScT: Standard care therapy; IQR: Interquartile range. Bold number: *p* Value ≤ 0.05.
